# Patient satisfaction with the Nigerian National Health Insurance Scheme two decades since establishment: A systematic review and recommendations for improvement

**DOI:** 10.4102/phcfm.v14i1.3003

**Published:** 2022-01-13

**Authors:** Onyemaechi Nwanaji-Enwerem, Paul Bain, Zoe Marks, Pamaji Nwanaji-Enwerem, Catherine A. Staton, Ayobami Olufadeji, Jamaji C. Nwanaji-Enwerem

**Affiliations:** 1 Harvard John F. Kennedy School of Government, Cambridge, Massachusetts, United States of America; 2 Duke University School of Medicine, Durham, North Carolina, United States of America; 3 Countway Library, Harvard Medical School, Boston, Massachusetts, United States of America; 4 Department of Business and Entrepreneurship, Barber-Scotia College, Concord, North Carolina, United States of America; 5 Division of Emergency Medicine, Duke University, Durham, North Carolina, United States of America; 6 Department of Emergency Medicine, Beth Israel Deaconess Medical Center, Harvard Medical School, Boston, Massachusetts, United States of America; 7 Gangarosa Department of Environmental Health, Emory Rollins School of Public Health and Department of Emergency Medicine, Emory University School of Medicine, Atlanta, Georgia, United States of America

**Keywords:** Nigeria, health insurance, patient satisfaction, systematic review, NHIS, SDG 2030

## Abstract

**Background:**

To improve healthcare access and mitigate healthcare costs for its population, Nigeria established a National Health Insurance Scheme (NHIS) in 1999. The NHIS remains Nigeria’s leading vehicle for achieving universal health coverage; nonetheless, questions remain regarding its quality and effectiveness. Studies on patient satisfaction have served as a useful strategy to further understand the patient experience and the efficacy of health systems.

**Aim:**

To synthesise current knowledge on patient satisfaction with the NHIS.

**Methods:**

The authors performed a systematic review of primary literature from 1999 to 2020 reporting on NHIS patient satisfaction in eight databases (including PubMed, Embase, and Africa-wide Information).

**Results:**

This search returned 764 unique records of which 21 met criteria for full data extraction. The 21 qualifying studies representing 11 of the 36 Nigerian states, were published from 2011 to 2020, and found moderate overall satisfaction with the NHIS (64%). Further, when disaggregated into specific domains, NHIS enrolees were most satisfied with provider attitudes (77%) and healthcare environments (70%), but less satisfied with laboratories (62%), billings (62%), pharmaceutical services (56%), wait times (55%), and referrals (51%). Importantly, time trends indicate satisfaction with the NHIS is increasing – although to differing degrees depending on the domain.

**Conclusion:**

The beneficiaries of the NHIS are moderately satisfied with the scheme. They consider it an improvement from being uninsured, but believe that the scheme can be considerably improved. The authors present two main recommendations: (1) shorter wait times may increase patient satisfaction and can be a central focus in improving the overall scheme, and (2) more research is needed across all 36 states to comprehensively understand patient satisfaction towards NHIS in anticipation of potential scheme expansion.

## Introduction

Lack of access to healthcare and unaffordable healthcare, remain major sources of hardship for households across the world. According to The World Health Organization (WHO), nearly 100 million people are being pushed into extreme poverty as a result of medical bills and over 930 million people spend 10% or more of their household income on healthcare services.^1^ As a result of the extreme burden related to healthcare costs, universal health coverage (UHC) has been included amongst the WHO 2030 Sustainable Development Goals (SDGs).^1^ The WHO advocated that member states pursue compulsory health insurance, more equitable tax systems, or a combination, as a path to reduce direct payments and user fees for health services.^2^ Towards the end of the 20th century, many African nations began initiating such reforms. Some nations adopted national health insurance schemes (NHIS) based on insurance contributions and tax funding. Others opted to remove user fees for priority health services or for targeted vulnerable groups.^3^

Nigeria’s present mechanism of achieving UHC, the Nigerian Health Insurance Scheme (NHIS), was established in 1999 under Act 35 of the Constitution and became operational in 2005. The NHIS is a pre-payment model where participants pay a fixed amount and the accumulated funds are pooled, allowing for participating Health Maintenance Organisations (HMOs) to pay for individuals needing medical care. The HMOs are responsible for purchasing healthcare services on behalf of registered enrolees. To ensure the most effective coverage for the population, the NHIS has specific programmes for different segments of society, but only the Formal Sector Health Insurance Programme (FSHIP) is functional.^4^ The FSHIP is further divided into three sub-sections comprising:

- public sector organisations (federal, state, local government officials, armed forces, police, and other uniformed services).
- private sector organisations (10 or more employees).
- group, individual, and family contributors (GIFSHIP) (e.g. small-scale enterprises with fewer than 10 staff, self-employed individuals, families and groups, retirees and retiree associations, diaspora groups, foreigners living in Nigeria, and adopted persons).

Like all insurance schemes, the NHIS is an insurance model that aims to mitigate the risks individuals bear by distributing the financial burden across the insured population pool. Payments to the NHIS are related to the earnings of the employee. For the public (Federal) sector programme, the employer pays 3.5% and the employee pays 1.75% of the employee salary into the scheme. For the private sector programme, the employer pays 10.0% whilst the employee pays 5.0% into the scheme.^5^ Individuals enrolled in GIFSHIP pay a fixed annual amount depending on the size of the group (NHIS).^6^ For both the public and private sector programmes, contributions made to the insurance scheme cover the employee, a spouse, and up to four biological children under the age of 18 years. Additional dependents or children require additional contributions by the insured. In order for enrolees to receive benefits, the employer must first register its organisation and the employee in the scheme. Following enrolment, the employer selects an HMO from the list of accredited HMOs. Finally, the employee registers him or her along with their dependents with a provider from a list of HMO-approved accredited healthcare providers. From the date of enrolment, there is a 90-day waiting period to access care and a minimum enrolment of six months to access high-cost drugs and surgical procedures. When fully enrolled in the NHIS, patients are entitled to receive provisions as outlined by the standard benefits package, which includes the following:^6^

- Inclusions:
  ■ Standard outpatient care and annual physicals.
  ■ Prescribed drugs included in the NHIS Drugs List (enrolee pays 10% of the cost).
  ■ Diagnostic tests that are included in the NHIS diagnostic test list.
  ■ Maternity care for up to four pregnancies ending in live births (additional care is provided for stillbirths; and live births are covered for an additional 12 weeks after the delivery date).
  ■ Preventative care and immunisations.
  ■ Specialist consults (e.g. orthopaedic surgeons, ear, nose and throat (ENT) surgeons, dental surgeons, ophthalmologists).
  ■ Inpatient hospital care up to 21 days per year.
  ■ Surgical procedures.
  ■ Emergency care.
  ■ Eye exam, eyeglasses (excluding contact lenses) up to N10 000.00.
  ■ Prostheses (limited to prosthesis produced in Nigeria).
  ■ Standard dental care.
- Exclusions:
  ■ Injuries related to occupational accidents.
  ■ Injuries from natural disasters (e.g. earthquakes, landslides, conflicts, wars, riots, social unrest).
  ■ Epidemics.
  ■ Family planning commodities (e.g. condoms).
  ■ Extreme sports related injuries (e.g. boxing, horse racing).
  ■ Drug abuse/addiction.
  ■ Home visits.
  ■ Mammoplasty.
  ■ Contact lenses.
  ■ Anti-tuberculosis (TB) drugs.
  ■ Treatment of congenital abnormalities requiring advanced surgical procedures (Tetralogy of Fallot, atrial septal defect, ventricular septal defect).
  ■ Artificial insemination (IVF).
  ■ Advanced dental care (crowns, bridges, bleaching, implants).
  ■ Post-mortem examinations.
- Partial exclusions:
  ■ High technology imaging (e.g. computed tomography [CT] scan and magnetic resonance imaging [MRI]: HMOs pay 50% of the cost).
  ■ Dialysis for acute renal failure (maximum of six sessions).

Surveys to understand patient satisfaction – the extent to which patients are happy with their healthcare – have become an essential feature of the patient experience and have also served as a tool to evaluate the quality and efficacy of healthcare systems.^7^ Amongst healthcare consumers (i.e. patients), satisfaction is multidimensional and depends on numerous factors, including the healthcare provider, cost, healthcare facility, and waiting times.^8^ Trends in patient satisfaction can inform policymakers, healthcare leaders, and non-patient actors (i.e. non-profit organisations, hospitals) on elements of healthcare that are working well or elements that need improvement. Patient satisfaction is also correlated with important outcome measures, including patient compliance with provider recommendations, utilisation of medical services, malpractice litigation, and overall prognosis.^9,10,11^ Furthermore, some studies have suggested that quality improvement measures that are informed from patient perspectives improve the safety, accessibility, and comprehensiveness of the care received.^12^ Given this context, for the overall Nigerian healthcare ecosystem, it is important to understand levels of overall and domain-specific satisfaction of NHIS enrolees. In the present study, the authors performed a systematic review of peer-reviewed literature to synthesise studies focusing on NHIS patient satisfaction. The authors then examined these works for trends to inform future efforts aimed at improving the NHIS.

## Methods

### Search strategy

Studies examining the satisfaction of patients with the Nigerian Health Insurance Scheme (NHIS were identified by searching the electronic databases PubMed (National Center for Biotechnology Information [NCBI]), Embase (Elsevier), Web of Science Core Collection (Clarivate), Global Index Medicus (World Health Organization [WHO]), EconLit (ProQuest), PAIS Index (ProQuest), Africa-wide Information (Elton B. Stephens CO [EBSCO]), and Global Health (EBSCO). The search was designed to capture all records representing English-language articles that considered national health insurance in the geographic context of Nigeria. Relevant controlled vocabulary terms were included when available (see Table 1). Searches were conducted on 31 October 2020, and were limited to articles published after 01 January 1999 (the NHIS was established by Act 35 of the Nigerian Constitution, adopted on 29 May 1999). The review management application Covidence (London, United Kingdom [UK]) was used to screen results.

**TABLE 1:** Electronic database searches.

Database	Search terms	Number of records
Africa-Wide Information (EBSCO)	(TI(“nigeria*”) OR AB(“nigeria*”) OR KW(“nigeria”)) AND (TI(“insurance scheme” OR “national insurance” OR “national health program*” OR “nhis”) OR AB(“insurance scheme” OR “national insurance” OR “national health program*” OR “nhis”) OR KW(“insurance scheme” OR “national insurance” OR “national health program*” OR “nhis”))	178
EconLit (ProQuest)	(su(nigeria) OR ti(nigeria*) OR ab(nigeria*) OR af(nigeria*)) AND (su(“Health Insurance, Public and Private (I13)”) OR ti(“insurance scheme” OR “national insurance” OR “national health program*” OR “nhis”) OR ab(“insurance scheme” OR “national insurance” OR “national health program*” OR “nhis”))	22
Embase (Elsevier; 1974)	(‘Nigeria’/exp OR nigeria*:ti,ab,kw,ad,ff,ca AND (‘health insurance’/de OR ‘national health insurance’/exp OR ‘public health insurance’/exp OR ‘social insurance’/exp OR ‘health insurance’:ab,ti,kw OR ‘insurance scheme’:ab,ti,kw OR ‘national insurance’:ab,ti,kw OR ‘national health program*’:ab,ti,kw OR ‘nhis’:ab,ti,kw)	370
Global Health (EBSCO)	(SU(nigeria) OR TI(“nigeria*”) OR AB(“nigeria*”)) AND (SU(“Health Insurance”) OR TI(“insurance scheme” OR “national insurance” OR “national health program*” OR “nhis”) OR AB(“insurance scheme” OR “national insurance” OR “national health program*” OR “nhis”))	149
Global Index Medicus, including African Index Medicus (WHO)	(tw:(nigeria*)) OR (pais_afiliacao:(nigeria)) OR (cp:(nigeria)) AND (tw:((“insurance scheme” OR “national insurance” OR “national health program*” OR “nhis”)))	29
PAIS Index (ProQuest)	(su(nigeria) OR ti(nigeria*) OR ab(nigeria*)) AND (su(“Health Insurance”) OR ti(“insurance scheme” OR “national insurance” OR “national health program*” OR “nhis”) OR ab(“insurance scheme” OR “national insurance” OR “national health program*” OR “nhis”))	21
Pubmed (NCBI)	(nigeria*[tw] OR nigeria*[ad]) AND (“Insurance, Health”[Mesh] OR “National Health Programs”[mesh] OR health insurance[tiab] OR insurance scheme[tiab] OR national insurance[tiab] OR national health program*[tiab] OR nhis[tiab])	338
Web of Science Core Collection (Clarivate)	(TS=”nigeria*” OR AD=”nigeria*” OR OO=”nigeria*” OR CU=”nigeria”) AND TS=(“insurance scheme” OR “national insurance” OR “national health program*” OR “nhis”)	119
Duplicates	-	−462
**Total**	**-**	**764**

Note: All searches were performed on 31 October 2020.

NHIS, National Health Insurance Scheme; WHO, World Health Organization; NCBI, National Center for Biotechnology Information; EBSCO, Elton B. Stephens CO (company); PAIS, Public Affairs International Service.

### Study selection

We included studies reporting on patient satisfaction with the NHIS. Studies utilising both qualitative and quantitative reporting methods were included. Additionally, to be included, studies must have met additional criteria of being full text (free and subscription), written in English, and peer-reviewed. Studies were excluded if they were non-English studies, if they did not evaluate patient satisfaction of the NHIS (this includes studies only reporting on provider [e.g. physician] satisfaction), if the study was a government/agency report, or if the work was a review, commentary, or other secondary studies. The authors of this study worked independently to evaluate eligible studies given the aforementioned criteria. In instances of coding disagreement, the authors discussed the items to reach a consensus. All identified works underwent title and abstract screening using Covidence. A subset of items seemingly meeting inclusion criteria was selected for full-text review. After full-text review, items were formally selected for final data extraction.

### Data extraction

Data was extracted using Covidence. Collected data included reported overall and domain-specific measures of satisfaction towards the NHIS, study authors/institutions, study design, study date, study inclusion/exclusion criteria, study region, and study participant demographic characteristics. The quality of each study was also assessed using the Joanna Briggs Institute’s (JBI) critical appraisal checklist for prevalence studies.^13^

### Statistical analysis

We calculated descriptive statistics for overall and domain-level satisfaction values. This included normal and weighted means based on study sample size. Additionally, the authors performed Spearman correlations to explore relationships between study year, study quality scores, satisfaction scores, and state populations based on 2006 Nigerian census data.^14^ All statistical analyses were performed using R version 3.6.3 (R Core Team, Vienna, Austria).

## Results

### Final study sample characteristics

**FIGURE 1:**
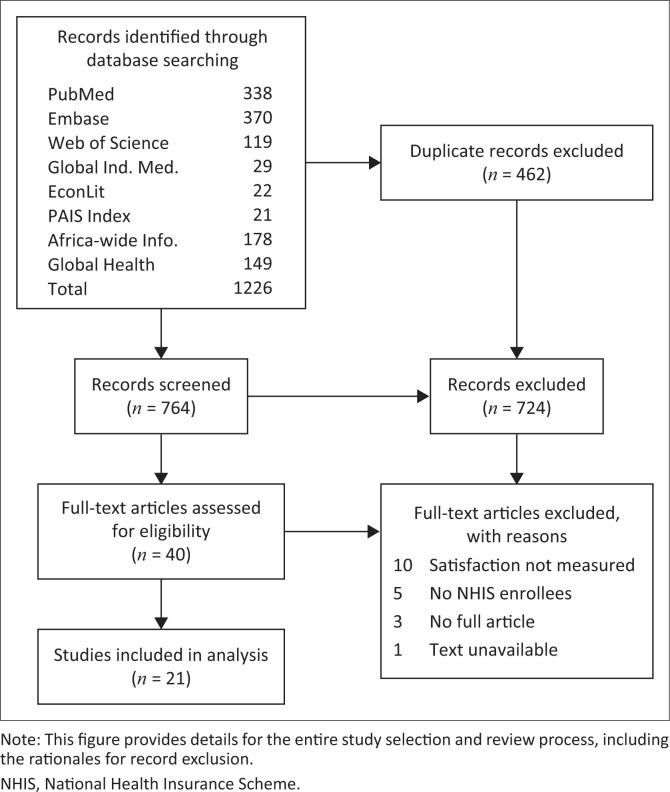
Study selection and review flow chart.

The search strategy identified 764 unique records. After initial screening, 40 full-text articles were examined in detail, 21 of which were eventually included in the final analysis (see Figure 1). All studies were cross-sectional in design, and on average were of good quality with a mean, range (standard deviation [s.d.]) quality score of 9.3, 7–10 (0.9). Most studies lost points for having smaller sample sizes or not describing subjects or the study setting in detail. Studies were published from 2011 to 2020 with a mean, range (s.d.) sample size of 381, 110–809 (198) participants. The 21 studies were conducted in 11 of 36 Nigerian states: Abia, Abuja (two studies), Enugu (two studies), Kaduna (three studies), Kano (three studies), Kwara (two studies), Lagos, Niger, Nasarawa, Plateau, and Oyo (four studies). Most of these states are in the western and central regions of Nigeria. Based on 2006 national census data, the 11 study states account for 35% of the total Nigerian population with Kano, Lagos, Kaduna, and Oyo being Nigeria’s four most populous states (see Table 2).

**TABLE 2:** Studies for data extraction.

Study/year	State	State population†	Characteristics			Satisfaction domains (%)							
			Sample size	Average age (years)	% females	Overall	Attitude (%)	Billing	Environment	Laboratories	Pharmacy	Referrals	Wait time
Abiola et al. ^30^	Lagos	9 113 605	487	34.10	54	96	-	-	-	-	-	-	-
Adewole et al. ^4^	Kwara	2 365 353	370	35.00	41	88	42	92	46	40	44	-	28
Adewole et al. ^33^	Oyo	5 580 984	373	42.50	44	53	93	-	-	-	83	83	59
Akande et al. ^19^	Kwara	2 365 353	210	40.78	68	58	90	40	-	-	17	-	-
Daramola et al. ^29^	Nasarawa	1 869 377	421	18–39†	43	63	61	-	60	72	55	-	57
Daramola et al. ^28^	Abuja/Federal Capital Territory	1 405 201	388	30–39§	47	58	-	-	62	67	54	-	59
Garba et al. ^23^	Kano	9 401 288	299	33.40	-	78	73	-	94	64	-	-	82
Ibrahim et al. ^15^	Niger	3 954 772	144	38.00	53	49	-	-	-	-	-	-	-
Iloh et al. ^16^	Abia	2 845 380	400	34.80	55	67	82	-	68	59	76	-	48
Kofoworola et al. ^20^	Abuja	1 405 201	279	35–44¶	49	75	91	52	-	85	68	-	51
Lar et al. ^27^	Plateau	3 206 531	400	30–49††	85	39	-	-	-	-	-	-	-
Michael et al. ^18^	Kano	9 401 288	202	36.40	49	66	84	78	-	76	77	-	30
Michael et al. ^25^	Kano	9 401 288	220	35.80	51	66	83	80	64	74	39	-	-
Mohammed et al. ^21^	Kaduna	6 113 503	280	42.49	18	42	-	-	-	-	-	-	-
Mohammed et al. ^22^	Kaduna	6 113 503	796	≥ 40.00‡‡	46	72	81	-	83	-	-	-	68
Mohammed et al. ^31^	Kaduna	6 113 503	796	> 29.00§§	46	64	-	-	-	-	-	-	-
Nwankwor et al. ^26^	Enugu	3 267 837	380	40.44	59	23	-	-	-	-	16	11	-
Oladimeji et al. ^34^	Oyo	5 580 984	311	37.10	46	80	80	-	83	-	51	-	-
Osungbade et al. ^32^	Oyo	5 580 984	333	42.50	47	84	52	38	-	-	56	59	78
Saka et al. ^24^	Oyo	5 580 984	110	55.49	38	64	93	-	-	21	91	-	50
Ujunwa et al. ^17^	Enugu	3 267 837	809	¶¶	48	57	-	55	-	-	-	-	47

† , From 2006 Nigeria National Census;

‡ , Majority of subjects 76.7%;

§ , Majority of subjects 39.7%;

¶ , Majority of subjects 36.6%;

†† , Majority of subjects 62.0%;

‡‡ , Majority of subjects 51.4%;

§§ , Majority of subjects 84.2%;

¶¶ , Age not mentioned in this study.

The studies had a good representation of both male and female participants with most participants being of middle age. The mean, range (s.d.) percentage of female and male study participants was 49.9%, 15% – 82.5% (12.7%) and 49.1%, 17.5% – 85% (12.8%), respectively. Mean participant ages ranged from 33.4 years to 55.49 years of age.

### Overall satisfaction score

All 21 studies reported satisfaction scores with the overall NHIS.^15,16,17,18^ Overall NHIS satisfaction scores had a mean, range (s.d.) of 64%, 23% – 96% (17%). The weighted mean for overall NHIS satisfaction scores was 65%. These findings were further contextualised by reports that 91.3% of respondents agreed that the NHIS is better than the previous system of medical care,^19^ and that 88.4% of respondents wanted the NHIS sustained and improved rather than a completely different alternative.^20^ Respondents also reported greater overall satisfaction if someone in their household had been sick in the past month.^21^

### Domains of satisfaction through the patient care journey

#### Healthcare environment satisfaction

Reporting on specific domains of satisfaction was less uniform amongst the studies. Beginning with the domain of healthcare environment, which NHIS enrolees first encounter when seeking care, eight studies reported significant findings related to the physical hospital and healthcare environment. The mean, range (SD) environment satisfaction score was 70%, 46% – 94% (16%). The weighted environment satisfaction mean was 71%. Work from Kaduna further suggested that there may be important differences in environment satisfaction depending on if an NHIS enrolee was visiting a public or private facility. In particular, the study reported that NHIS enrolees receiving care from private providers were more likely to perceive better facility quality than those receiving care from public providers.^22^

#### Wait time and referral satisfaction

After arriving at a healthcare facility, NHIS enrolees are likely to spend some time waiting before they can be attended to by a healthcare provider. Twelve studies reported wait time related satisfaction scores with a mean, range (SD) score of 55%, 28% – 82% (16%), suggesting significant variation and relatively high dissatisfaction levels. The weighted wait time satisfaction mean was 56%. Similar to environment satisfaction, there were notable differences in wait times depending on if an enrolee was being treated at a public or private facility. In a study assessing promptness of care, insured users were significantly less likely to express that they received prompt attention when receiving care from public providers as compared to when receiving care from private providers.^22^ Males in the study also reported significantly greater promptness of care when compared to females.^22^ Two studies where NHIS enrolees were compared to uninsured persons provide added context to these wait time satisfaction scores. In the first study, insured patients perceived an improvement in waiting times since obtaining insurance and were more satisfied (82%) with their overall time spent in the hospital when compared to the uninsured (66%).^23^ This suggests some success on the part of the NHIS. However, the second study seemingly offered a contradictory trend given reports that a greater proportion of the insured (50%) reported issues with waiting time when compared to the uninsured (6%).^24^ Nonetheless, these authors also reported that some care providers spent more time documenting aspects of care of the insured compared to the uninsured.

A final element of assessing wait time satisfaction involves understanding how an enrolee arrived at a facility. In other words, did the enrolee arrive on his or her own behest or was the enrolee referred? Enrolees who received secondary or tertiary referrals were more likely to report prompt attention when compared to those receiving no referrals.^22^ Three studies explicitly presented referral satisfaction scores. Those studies had a mean, range (SD) referral score of 51%, 11% – 83% (37%). Referral satisfaction had a weighted mean score of 50%.

#### Provider attitude satisfaction

After waiting for some time, the enrolee next meets the healthcare provider. Thirteen studies presented satisfaction scores related to provider attitudes with a mean, range (s.d.) score of 77%, 42% – 93% (16%). Provider attitudes had a weighted mean score of 76%. The reviewed studies demonstrated that the number of providers could influence satisfaction. For instance, a Kano state study reported that satisfaction with the available number of doctors was associated with an almost two times greater odds of overall satisfaction.^25^ On the contrary, 88.7% of the respondents in the study in Enugu state felt that facilities were staffed with adequate numbers of personnel, but 79.5% of the study sample still reported poor health services.^26^ A Kaduna state study further characterised service into four subdomains^22^: (1) dignity and respectful treatment, (2) communication clarity, (3) autonomy in decision making, and (4) patient choice of provider. In comparisons of enrolees receiving care from public and private providers, those receiving care from public providers were more likely to report better communication with the provider, but less likely to feel that they were being treated with dignity/respect, less likely to report autonomy in decision making, and less likely to report having choice of providers.^22^

#### Pharmacy and lab satisfaction

After being seen by a provider, enrolees are next likely to encounter the pharmacy or laboratory if deemed necessary. Thirteen studies reported pharmacy-related satisfaction scores with a mean, range (s.d.) score of 56%, 16% – 91% (23%). The weighted pharmacy satisfaction mean was 55%. Findings from a Kano study further contextualised these results through reports of 60% of their study participants being dissatisfied with explanations for drug unavailability, but 90% being satisfied with explanations on how to use medications.^25^ Nine studies reported findings on satisfaction in the domain of laboratory services with a mean, range (s.d.) score of 62%, 21% – 85% (20%). Laboratories had a weighted satisfaction mean of 64%.

#### Billing satisfaction

At the end of their care encounter, enrolees must tender payments for services received. Seven studies presented satisfaction scores related to billing, costs, and payments with a mean, range (s.d.) score of 62%, 38% – 92% (21%). Billing had a weighted mean satisfaction score of 61%. Furthermore, a Plateau study found that many participants were dissatisfied with payments because of inconsistent prices for pharmaceuticals and a frequent reliance on out-of-pocket expenditures despite enrolment in the NHIS.^27^ Continuously incurring additional costs despite NHIS enrolment was echoed by findings from a study conducted in Kano state, which reported significant relationships between spending less in out-of-pocket expenditures and higher satisfaction.^23^ Nonetheless, median out-of-pocket expenditures were lower for enrolees; specifically for uninsured and insured persons at their last visits, these costs were N2100.00 (Nigerian naira) and N1200.00, respectively.^23^ This difference was statistically significant similar to the mean (s.d.) monthly pre- and post-NHIS enrolment health expenditures reported from Kwara state: N3040.40 (2552.80) pre-NHIS enrolment versus N782.20 (637.40) post-NHIS enrolment.^19^

### Temporal, quality, population, and geographical trends

**FIGURE 2:**
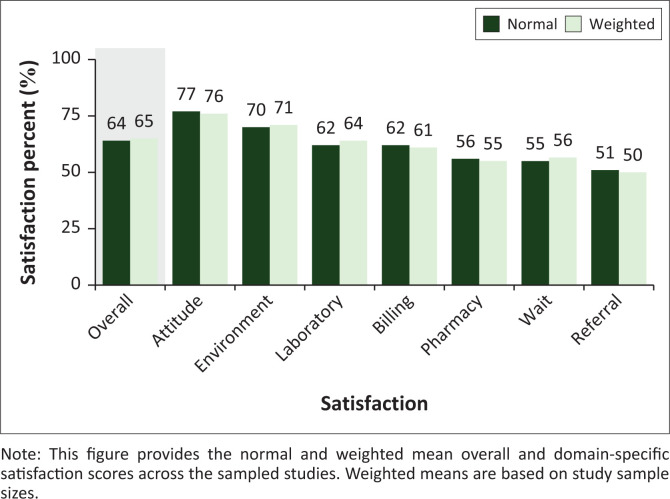
Mean satisfaction scores.

**FIGURE 3:**
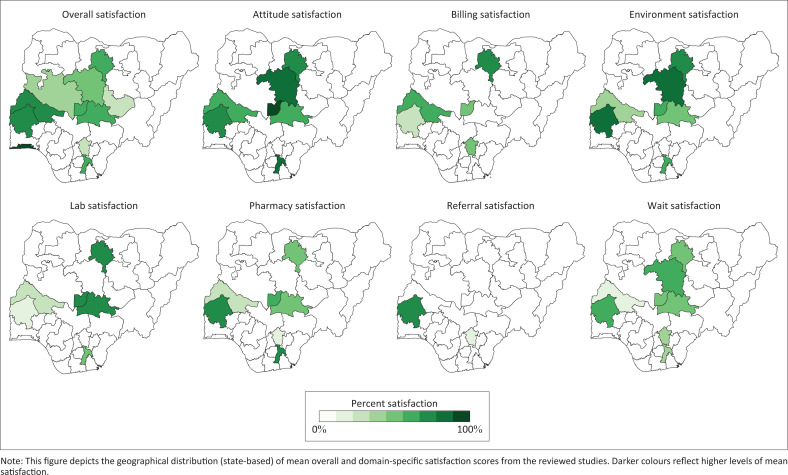
Geographic distribution of satisfaction scores.

Satisfaction scores were highest for provider attitudes (77%) and lowest for referrals (51%) (see Figure 2). Provider attitude and environment satisfaction scores were the only domains with values higher than the overall satisfaction score. From largest to smallest, Spearman correlation coefficients for year of publication and satisfaction were: referrals (*r* = 0.50), billing (*r* = 0.44), provider attitude (*r* = 0.32), overall (*r* = 0.29), laboratories (*r* = 0.29), environment (*r* = 0.23), pharmacy (*r* = 0.22), and wait times (*r* = 0.18). Although they cannot speak to causal relationships, these results suggest that referrals, billing, and provider attitude satisfaction have improved the most with time whilst wait time satisfaction has improved the least. Again, from largest to smallest, Spearman correlation coefficients for state population and satisfaction were: referrals (*r* = 0.87), environment (*r* = 0.71), wait times (*r* = 0.25), overall (*r* = 0.23), billing (*r* = 0.22), pharmacy (*r* = 0.20), provider attitude (*r* = 0.03), and laboratories (*r* = –0.09). These results suggest that more populous states have higher referral and environment satisfaction, but also have poor laboratory satisfaction. Figure 3 displays geographical trends in overall and domain-specific satisfaction scores.

### Satisfaction score and demographic characteristics

#### Age relationships

With respect to relationships between age and overall NHIS satisfaction score or a related domain, two studies reported approximately 4% higher average satisfaction scores in their older participants compared to the younger participants.^21,28^ In the Kaduna state study, mean ages for older and younger participants were 44.4 years and 41.1 years, respectively.^21^ In the Abuja-based study, participants < 30 years were compared to those aged 50–60 years.^28^ In contrast, a Nasarawa state study reported an approximately 5.6% higher satisfaction in younger people (< 40 years) compared to their older counterparts.^29^

Related to this, there is evidence to suggest that some of the relationships between age and satisfaction were related to healthcare utilisation, family coverage, professional rank, and duration of enrolment. Specifically, there were significant relationships between age and NHIS utilisation with older people (mean age 39.6 years) having higher NHIS utilisation than their counterparts (mean age 33.5 years).^30^ Furthermore, young enrolees had a higher likelihood of having all of their family members insured through the NHIS. More specifically, individuals with greater than four children reported lower NHIS coverage of their family members than their peers with at most four children.^31^

Professional rank is often associated with age and also demonstrated important relationships with satisfaction in the reviewed studies. Two studies in Kaduna and Kwara states found that junior staff persons were more satisfied with the NHIS than their senior counterparts by at least 13%.^19,21^ Studies also reported specific data on enrolment duration relationships.^19,23,32^ Two Kano and Kwara state studies reported 8% higher satisfaction in persons with shorter enrolment duration. In the Kwara study, shorter duration was defined as being enrolled for less than 12 months.^19^ In the Kano study, the median enrolment duration of satisfied and unsatisfied respondents was one year and four years, respectively.^23^ An Oyo-based study reported the opposite, with participants with five years of enrolment demonstrating significantly higher satisfaction scores by approximately 11% when compared to their peers enrolled for four or fewer years.^32^ Mohammed et al. reported on additional important enrolment trends. In particular, in their study, enrolees with more than two years of enrolment were less likely to have all of their family members insured and less likely to have better access to benefits packages for treatments compared to their peers with at most two years enrollment.^31^

#### Sex and marital status relationships

An Abuja and Plateau state study reported significantly higher satisfaction scores of approximately 20% in males compared to females.^27,29^ A later Oyo state study reported the opposite with females having 10% higher satisfaction.^33^ Single, divorced, or widowed respondents in Kaduna were reported as having lower satisfaction than respondents with polygamous status.^21^ Similar to previous findings with age, one study observed a significant relationship between marital status and NHIS utilisation with married people having higher levels of utilisation.^30^

#### Education relationships

Six studies observed significant relationships with education and satisfaction scores. Two studies in Nasarawa and Kaduna found an inverse relationship between education and satisfaction. The Nasarawa study reported that compared to those with primary, secondary, and tertiary education, persons with no formal education had at least 23% higher levels of satisfaction with the NHIS.^29^ The Kaduna study found that participants with an education below tertiary level were 13% more satisfied than their peers.^21^ However, when they asked about specific knowledge about health insurance and awareness of money contributions in health insurance, they found that participants with less knowledge and awareness were at least 25% less satisfied. A later study by the same Kaduna study authors, reported that NHIS enrolees with less education reported higher coverage of their family members compared to their peers with more education.^31^ Work in Abuja and Oyo reported higher satisfaction with higher education attainment.^28,32^ Education also played a role in enrolees choosing to forgo treatment at closer facilities. Bypassing behaviour was most prevalent in participants with tertiary education and resulted in additional expenditures of time (mean [s.d.] = 35.1 [34.7] min) and money (mean [s.d.] = N389.51 [545.21]) for healthcare.^34^

## Discussion

The NHIS remains Nigeria’s leading mechanism for achieving the WHO’s 2030 SDG of UHC, although it remains far from achieving this goal. Approximately two-thirds of respondents were satisfied with the NHIS. An even greater number of respondents believed that the scheme was better than past coverage mechanisms and should be improved rather than substituted for an alternative. Yet, because the scheme only serves about 4% of the Nigerian population, there remains a glaring need for expansion.^35^ Nevertheless, it remains important to optimise the scheme in concert with efforts directed at expansion. Further support for this rationale comes from other schemes across Africa. Ghana is an example of a nearby African nation with a national health insurance scheme that covers a substantially higher proportion of its total population (38%), and has a satisfaction score of 76%.^36^ Despite covering a larger proportion of its population, Ghana continues to face issues in the domains of wait times, medications, and provider attitudes.^37^ Hence, understanding how to improve overall and specific domains of satisfaction will likely be of considerable utility both pre- and post-expansion of the NHIS in Nigeria.

Identifying specific deficits in NHIS patient satisfaction was a major motivation for this study. Given the well-documented relationships of patient satisfaction with overall healthcare system quality,^7^ targeted interventions have the potential of improving specific domains of patient satisfaction and the health system at large. Understanding relationships of demographic factors with satisfaction helps in laying the groundwork for targeted interventions because these relationships help in understanding which specific persons are not satisfied. In this review, the authors identified mixed findings on the relationships of overall and domain-specific satisfaction with demographic characteristics like age and sex. Although there was no consensus on whether variables like age were associated with increased or decreased satisfaction, evidence suggests that these demographic relationships were meaningfully impacted by other factors like NHIS utilisation, family coverage, and enrolment duration. For instance, one study documented that respondents were more satisfied if they had a sick household member in the past month.^21^ Thus, understanding that older people often have greater NHIS utilisation as a result of comorbidities can better contextualise their relationship with satisfaction. Given their greater need for care, they may be more relieved to have NHIS coverage and therefore tend to be more satisfied. Similarly, a seemingly contradictory study reporting that younger people are more satisfied with the NHIS can be better contextualised by understanding that younger people may have smaller families. Therefore, more, if not all, of their family members are likely to be covered by the NHIS, resulting in more favourable satisfaction levels. Hence, to be most effective, the findings suggest that instead of simply focusing on age, interventions should shift their focus to identifying other characteristics such as better serving individuals with greater health needs or with family sizes where coverage needs are not being met.

A similar lesson can be taken from the authors’ observed education and satisfaction relationships. Education relationships demonstrated complexity with higher levels of education being associated with increased and decreased NHIS satisfaction. As is common in the literature, education can be viewed as a measure of socioeconomic status which can have an important impact on health behaviors.^38^ For example, amongst the reviewed studies, people with more education were more likely to exhibit choice and bypassing behaviours.^34^ Even if it required more money and time, these individuals preferred to invest these additional resources to see their providers of choice. The authors also observed trends related to education and socioeconomic status in the data reporting on satisfaction based on care received by public or private providers. Persons receiving care from private providers often had higher satisfaction. Moreover, worse wait times, worse facility environments, and worse feelings of choice and autonomy in decision making were reported with public providers.^22^ Still, one of the most meaningful findings related to education was linked to NHIS-specific education. Specifically, individuals who were more knowledgeable about the NHIS and how it works were more satisfied by it.^21^ This phenomenon is not new as it has been documented by a number of previous studies. One study of a community-based insurance scheme in southwest Ethiopia reported that good knowledge of the insurance scheme was associated with a two times greater odds of being satisfied by the scheme.^39^ These authors describe poor knowledge of health insurance schemes as being associated with dropout and poor compliance. They also suggest communication and behavioural education as tools to overcome this problem. Nonetheless, it remains important to highlight such findings because educating enrolees about their insurance plan can represent a targeted intervention. These findings suggest that simply enrolling individuals is not enough; individuals must also be educated about their insurance plan and how to make the most of it. Additionally, working to first target such interventions to the public provider space may be of greatest benefit to those in most immediate need.

Central to the review and potential targeted efforts to improve the NHIS are the relationships of overall and domain-specific satisfaction scores with time. All of the correlations between the year of study publication and satisfaction scores were positive suggesting that satisfaction as a whole and its specific domains are improving with time. Nevertheless, the magnitude of this suggested improvement differed. For instance, when comparing the maximum and minimum correlations, the authors observed that satisfaction for referrals was almost three times higher than satisfaction for wait times. Furthermore, wait times having the lowest correlation may signal that it is amongst the greatest burdens still experienced by NHIS enrolees. Given that wait times are also tied into every aspect of care, it represents a focus where solutions could again have a profound impact. The healthcare literature on wait times is robust, including studies of care facilities in Africa.^40,41,42^ Several factors including patient visit patterns, patient expectations, and clinic practices can affect wait times.^43,44^ Hence, performing clinic-specific assessments can often prove useful for identifying wait time bottlenecks. Once identified, these bottlenecks can be addressed in a tailored manner. With respect to relationships with state population, which can have important implications on resource availability, environment and referral satisfaction scores were the highest-rated domains with scores at least two times greater than wait times and the other domains of satisfaction. This provides added impetus for these lower-ranked domains being emphasised as targets for solution building.

### Limitations of the study

Although this study provides useful insights regarding patient satisfaction with the NHIS, it does have some limitations. Firstly, although all reviewed studies reported an overall satisfaction score, the authors’ analysis presents an incomplete picture of NHIS satisfaction because not all states are represented and reporting for various domains of satisfaction was not unanimous. Nonetheless, the authors present all available data with attention to national geography to help highlight where future studies are particularly needed. Secondly, given that being a beneficiary of the presently functioning NHIS is predicated on being a formal sector employee, the authors’ findings may not be generalisable to all Nigerians, especially those primarily employed in the informal sector who constitute a majority of the working population. This issue of generalisability may be further complicated by variations/heterogeneity in study methods such as the exclusion of hospital employees. The authors anticipate that expanding NHIS programmes to the informal sector and other groups would produce similar satisfaction measurements; however, future studies are necessary to confirm these hypotheses. Thirdly, acutely ill people were often excluded from the reviewed studies and may have different perspectives on satisfaction. This is especially important given that serious or complex procedures and aspects of care (e.g. certain surgeries) may not be covered by the NHIS. Hence, the findings are limited to primary care and other elements of the Nigerian healthcare system presently included in the NHIS. Finally, limiting the search to articles in English could lead to reporting bias. Nevertheless, the authors do not believe that this is a major issue given that English is Nigeria’s national language and the subject matter is Nigeria specific.

## Conclusion

In this systematic review, we find that NHIS enrolees are moderately satisfied with the overall scheme (64%), and that satisfaction is increasing with time. Enrolees were most satisfied with provider attitudes (77%) and healthcare environments (70%), but least satisfied with laboratories (62%), billings (62%), pharmaceutical services (56%), waiting times (55%), and referrals (51%). From the authors’ results, it is clear that NHIS beneficiaries consider the scheme to be an improvement from prior insurance mechanisms or simply being uninsured. Still, there are just as many individuals who believe the scheme can be considerably improved. The authors’ findings suggest that by focusing on specific NHIS domains such as wait times, laboratory, and pharmacy services, meaningful advancements can be made by those hoping to improve the NHIS and Nigeria’s greater healthcare system. Nevertheless, there remain important considerations including awareness, geopolitical tensions, and resource capacity limits that individuals and organisations must consider as they engage in solution building. Further studies on the NHIS and reporting on improvement-oriented solutions will be invaluable for translating what is presently tremendous potential to healthier lives for all Nigerian citizens.

## References

[1] WHO. Universal health coverage (UHC) [homepage on the Internet]. 2019 [cited 2021 Feb 17]. Available from: https://www.who.int/news-room/fact-sheets/detail/universal-health-coverage-(uhc)

[2] Sanogo NA, Fantaye AW, Yaya S. Universal health coverage and facilitation of equitable access to care in Africa. Front Public Health. 2019;7:102. 10.3389/fpubh.2019.00102 31080792 PMC6497736

[3] Atim C. Second conference of the African Health economics and policy association: Towards universal healthcare coverage in Africa. Expert Rev Pharm Out Res. 2011 Jun 1;11(3):267–71. 10.1586/erp.11.20 21671694

[4] Adewole DA, Osungbade KO. Nigeria National Health Insurance Scheme: A highly subsidized health care program for a privileged few. Int J Trop Dis Health. 2016;19(3):1–11. 10.9734/IJTDH/2016/27680

[5] NHIS. Formal sector social health insurance programme (FSSHIP) – National Health Insurance Scheme [homepage on the Internet]. 2021 [cited 2021 Feb 17]. Available from: https://www.nhis.gov.ng/formal-sector-social-health-insurance-programmefsship/

[6] NHIS. Operations of the group, individual, and family social health insurance programme (GIFSHIP): A pamphlet for contributors [homepage on the Internet]. 2020 [cited 2021 Apr 13]; p. 1–22. Available from: https://www.nhis.gov.ng/?media_dl=2765

[7] Prakash B. Patient satisfaction. J Cutan Aesthet Surg. 2010;3(3):151–155. 10.4103/0974-2077.74491 21430827 PMC3047732

[8] Batbaatar E, Dorjdagva J, Luvsannyam A, Savino MM, Amenta P. Determinants of patient satisfaction: A systematic review. Perspect Public Health. 2017;137(2):89–101. 10.1177/1757913916634136 27004489

[9] Doyle C, Lennox L, Bell D. A systematic review of evidence on the links between patient experience and clinical safety and effectiveness. BMJ Open. 2013;3(1):e001570. 10.1136/bmjopen-2012-001570 PMC3549241 23293244

[10] Kennedy GD, Tevis SE, Kent KC. Is there a relationship between patient satisfaction and favorable outcomes? Ann Surg. 2014;260(4):592–600. 10.1097/SLA.0000000000000932 25203875 PMC4159721

[11] Prabhu KL, Cleghorn MC, Elnahas A, et al. Is quality important to our patients? The relationship between surgical outcomes and patient satisfaction. BMJ Qual Saf. 2018;27(1):48–52. 10.1136/bmjqs-2017-007071 29101291

[12] Xesfingi S, Vozikis A. Patient satisfaction with the healthcare system: Assessing the impact of socio-economic and healthcare provision factors. BMC Health Serv Res. 2016;16:94. 10.1186/s12913-016-1327-4 26979458 PMC4793546

[13] Munn Z, Moola S, Riitano D, Lisy K. The development of a critical appraisal tool for use in systematic reviews addressing questions of prevalence. Int J Health Policy Manag. 2014;3(3):123. 10.15171/ijhpm.2014.71 25197676 PMC4154549

[14] Nigeria Data Portal. State population, 2006 [homepage on the Internet]. 2006 [cited 2021 Mar 14]. Available from: https://nigeria.opendataforafrica.org/ifpbxbd/state-population-2006

[15] Ibrahim SA, Aliyu AA. Knowledge, attitude, perception and clients’ satisfaction with national health insurance scheme (NHIS) services at general hospital, Minna-Niger state-Nigeria. 13th World Congress on Public Health [April 23–27, 2012, Addis Ababa, Ethiopia]. 2012; p. 27.

[16] Iloh GUP, Ofoedu JN, Njoku PU, Odu FU, Ifedigbo CV, Iwuamanam KD. Evaluation of patients’ satisfaction with quality of care provided at the National Health Insurance Scheme clinic of a tertiary hospital in South-Eastern Nigeria. Niger J Clin Pract. 2012;15(4):469–474. 10.4103/1119-3077.104529 23238200

[17] Ujunwa FA, Onwujekwe O, Chinawa JM. Health services utilization and costs of the insured and uninsured under the formal sector social health insurance scheme in Enugu metropolis South East Nigeria. Niger J Clin Pract. 2014;17(3):331–335. 10.4103/1119-3077.130235 24714012

[18] Michael GC, Suleiman HH, Grema BA, Aliyu I. Assessment of level of satisfaction of national health insurance scheme enrolees with services of an accredited health facility in Northern Nigerian. Ann Trop Med Public Health. 2017;10(5):1271. 10.4103/ATMPH.ATMPH_372_17

[19] Akande TM, Salaudeen AG, Babatunde OA, et al. National health insurance scheme and its effect on staff’s financial burden in a Nigerian tertiary health facility. Int J Asian Soc Sci. 2012;2(12):2175–2185.

[20] Kofoworola AA, Ekiye A, Motunrayo AO, Adeoye AT, Adunni MR. National Health Insurance Scheme: An assessment of service quality and clients’ dissatisfaction. Ethiop J Health Sci. 2020;30(5):20. 10.4314/ejhs.v30i5.20 PMC8047258 33911842

[21] Mohammed S, Sambo MN, Dong H. Understanding client satisfaction with a health insurance scheme in Nigeria: Factors and enrollees experiences. Health Res Policy Syst. 2011;9(1):1–8. 10.1186/1478-4505-9-20 21609505 PMC3129583

[22] Mohammed S, Bermejo JL, Souares A, Sauerborn R, Dong H. Assessing responsiveness of health care services within a health insurance scheme in Nigeria: Users’ perspectives. BMC Health Serv Res. 2013;13(1):1–13. 10.1186/1472-6963-13-502 24289045 PMC4220628

[23] Garba MR, Gadanya MA, Iliyasu Z, Gajida AU. Comparative study of patients’ satisfaction between national health insurance scheme-insured and un-insured patients attending a Northern Nigerian tertiary hospital. Niger J Basic Clin Sci. 2018;15(2):118. 10.4103/njbcs.njbcs_48_16

[24] Saka SA, Fajemirokun OT. The effects of National Health Insurance Scheme on equity and quality of diabetes care in secondary healthcare facilities in SouthWest Nigeria. J Med Biomed Sci. 2018;7(1):11–21. 10.4314/jmbs.v7i1.2

[25] Michael GC, Aliyu I, Grema BA, Thacher TD. Impact of structural and interpersonal components of health care on user satisfaction with services of an outpatient clinic of a Nigerian tertiary hospital. Trop J Med Res. 2017;20:139–148. 10.4103/tjmr.tjmr_22_17

[26] Nwankwor C, Aneke C, Henry-Arize I, Okoronkwo I. Perceived utilization of National Health Insurance among Staff of University of Nigeria Teaching Hospital (UNTH), Ituku – Ozalla, Enugu, Southeast Nigeria. Int J Med Health Develop. 2018;23(2):255.

[27] Lar LA, Mafwalal BM, Ozoilo JU, Dakum LB, Ode GN. Participation in the National Health Insurance Scheme among nurses in a tertiary teaching hospital, North Central Nigeria. J Community Med Prim Health Care. 2012;24(1–2):69–73.

[28] Daramola OE, Adeniran A, Akande TM. Patients’ satisfaction with the quality of services accessed under the National Health Insurance Scheme at a tertiary health facility in FCT Abuja, Nigeria. J Community Med Prim Health Care. 2018;30(2):90–97.

[29] Daramola OE, Maduka WE, Adeniran A, Akande TM. Evaluation of patients’ satisfaction with services accessed under the National Health Insurance Scheme at a tertiary health facility in North Central, Nigeria. J Community Med Prim Health Care. 2017;29(1):11–17.

[30] Abiola AO, Ladi-Akinyemi TW, Oyeleye OA, Oyeleke GK, Olowoselu OI, Abdulkareem AT. Knowledge and utilisation of National Health Insurance Scheme among adult patients attending a tertiary health facility in Lagos State, South-Western Nigeria. Afr J Prim Health Care Fam Med. 2019;11(1):e1–e7. 10.4102/phcfm.v11i1.2018 PMC6779984 31588768

[31] Mohammed S, Aji B, Bermejo JL, Souares A, Dong H, Sauerborn R. User experience with a health insurance coverage and benefit-package access: Implications for policy implementation towards expansion in Nigeria. Health Policy Plann. 2016;31(3):346–355. 10.1093/heapol/czv068 26261105

[32] Osungbade KO, Obembe TA, Oludoyi A. Users’ satisfaction with services provided under National Health Insurance Scheme in south western Nigeria. Int J Trop Dis Health. 2014;4(5):595–607. 10.9734/IJTDH/2014/7280

[33] Adewole DA, Adeniji FIP, Adegbrioye SE, Dania OM, Ilori T. Enrollees’ knowledge and satisfaction with National Health Insurance Scheme service delivery in a tertiary hospital, South West Nigeria. Niger Med J. 2020;61(1):27–31. 10.4103/nmj.NMJ_126_18 32317818 PMC7113822

[34] Oladimeji AO, Adewole DA, Adeniji F. The bypassing of healthcare facilities among National Health Insurance Scheme enrollees in Ibadan, Nigeria. Int Health. 2020;13(3):291–296. 10.1093/inthealth/ihaa063 PMC8079309 32986116

[35] Enabulele O. Achieving universal health coverage in Nigeria: Moving beyond annual celebrations to concrete address of the challenges. World Med Health Policy. 2020;12(1):47–59. 10.1002/wmh3.328

[36] Dalinjong PA, Laar AS. The national health insurance scheme: Perceptions and experiences of health care providers and clients in two districts of Ghana. Health Econ Rev. 2012;2(1):13. 10.1186/2191-1991-2-13 22828034 PMC3505458

[37] Alhassan RK, Nketiah-Amponsah E, Arhinful DK. A review of the National Health Insurance Scheme in Ghana: What are the sustainability threats and prospects? PLoS One. 2016;11(11):e0165151. 10.1371/journal.pone.0165151 27832082 PMC5104458

[38] Pampel FC, Krueger PM, Denney JT. Socioeconomic disparities in health behaviors. Annu Rev Sociol. 2010;36:349–370. 10.1146/annurev.soc.012809.102529 21909182 PMC3169799

[39] Kebede KM, Geberetsadik SM. Household satisfaction with community-based health insurance scheme and associated factors in piloted Sheko district, Southwest Ethiopia. PLoS One. 2019;14(5):e0216411. 10.1371/journal.pone.0216411 31083706 PMC6513074

[40] Bruijns SR, Wallis LA, Burch VC. Effect of introduction of nurse triage on waiting times in a South African emergency department. Emerg Med J. 2008;25(7):395–397. 10.1136/emj.2007.049411 18573946

[41] Sastry A, Long KNG, Sa A De, et al. Collaborative action research to reduce persistently long patient wait times in two public clinics in Western Cape, South Africa. Lancet Glob Health. 2015;3:S18. 10.1016/S2214-109X(15)70137-3

[42] Owen MD, Floyd L, Bryce F, et al. Implementation of a triage system to reduce wait time and prioritise care for high-risk obstetric patients in a regional hospital in Accra, Ghana. Lancet Glob Health. 2016;4:S16. 10.1016/S2214-109X(16)30021-3

[43] Naidoo L, Mahomed OH. Impact of lean on patient cycle and waiting times at a rural district hospital in KwaZulu-Natal. Afr J Prim Health Care Fam Med. 2016;8(1):9. 10.4102/phcfm.v8i1.1084 PMC4969513 27543283

[44] Chu H, Westbrook RA, Njue-Marendes S, Giordano TP, Dang BN. The psychology of the wait time experience – What clinics can do to manage the waiting experience for patients: A longitudinal, qualitative study. BMC Health Serv Res. 2019;19(1):459. 10.1186/s12913-019-4301-0 31286957 PMC6615172

